# Boerhaave syndrome due to excessive alcohol consumption: two case reports

**DOI:** 10.1186/s12245-020-00318-5

**Published:** 2020-11-30

**Authors:** Yuichiro Haba, Shungo Yano, Hikaru Akizuki, Takashi Hashimoto, Toshio Naito, Naoyuki Hashiguchi

**Affiliations:** 1 Department of General Medicine, Juntendo University School of Medicine, 2-1-1 Hongo, Bunkyo-ku, Tokyo, 113-8421 Japan; 2 Department of Emergency and Disaster Medicine, Juntendo University School of Medicine, 2-1-1 Hongo, Bunkyo-ku, Tokyo, 113-8421 Japan; 3 Department of Esophageal and Gastroenterological Surgery, Juntendo University School of Medicine, Tokyo, Japan

**Keywords:** Boerhaave syndrome, Mallory–Weiss syndrome, Chest pain, Vomiting, Hematemesis

## Abstract

**Background:**

Spontaneous esophageal rupture, or Boerhaave syndrome, is a fatal disorder caused by an elevated esophageal pressure owing to forceful vomiting. Patients with Boerhaave syndrome often present with chest pain, dyspnea, and shock. We report on two patients of Boerhaave syndrome with different severities that was triggered by excessive alcohol consumption and was diagnosed immediately in the emergency room.

**Case presentation:**

The patient in case 1 complained of severe chest pain and nausea and vomited on arrival at the hospital. He was subsequently diagnosed with Boerhaave syndrome coupled with mediastinitis using computed tomography (CT) and esophagogram. An emergency operation was successfully performed, in which a 3-cm tear was found on the left posterior wall of the distal esophagus. The patient subsequently had anastomotic leakage but was discharged 41 days later. The patient in case 2 complained of severe chest pain, nausea, vomiting, and hematemesis on arrival. He was suggested of having Boerhaave syndrome without mediastinitis on CT. The symptoms gradually disappeared after conservative treatment. Upper gastrointestinal endoscopy performed on the ninth day revealed a scar on the left wall of the distal esophagus. The patient was discharged 11 days later. In addition to the varying severity between the cases, the patient in case 2 was initially considered to have Mallory–Weiss syndrome.

**Conclusion:**

Owing to similar histories and symptoms, Boerhaave syndrome and Mallory–Weiss syndrome must be accurately distinguished by emergency clinicians. CT can be a useful modality to detect any severity of Boerhaave syndrome and also offers the possibility to distinguish Boerhaave syndrome from Mallory–Weiss syndrome.

## Background

Boerhaave syndrome was first reported by Hermann Boerhaave in 1724 as a case of esophageal rupture caused by vomiting after a large meal [1]. It is induced by increased esophageal pressure followed by straining, which can result from retching, vomiting, weightlifting, childbirth, or defecation [2]. It is generally difficult to promptly diagnose Boerhaave syndrome, and patients can have fatal consequences if not intervened early [3]. It is also crucial to correctly distinguish Boerhaave syndrome from Mallory–Weiss syndrome as they share similar etiologies and initial clinical manifestations; however, they differ with respect to the site and depth of laceration, treatment administered, and prognosis. Commonly, patients with Boerhaave syndrome present with transmural lacerations to the left wall of the distal esophagus, requiring surgery for repair and reinforcement of ruptured wounds, irrigation, and drainage [3]. Conversely, patients with Mallory–Weiss syndrome have lacerations to the submucosal layer of the gastric cardia, warranting good prognoses with endoscopic hemostases or conservative therapies.

## Case presentation

### Case 1

**Fig. 1:**
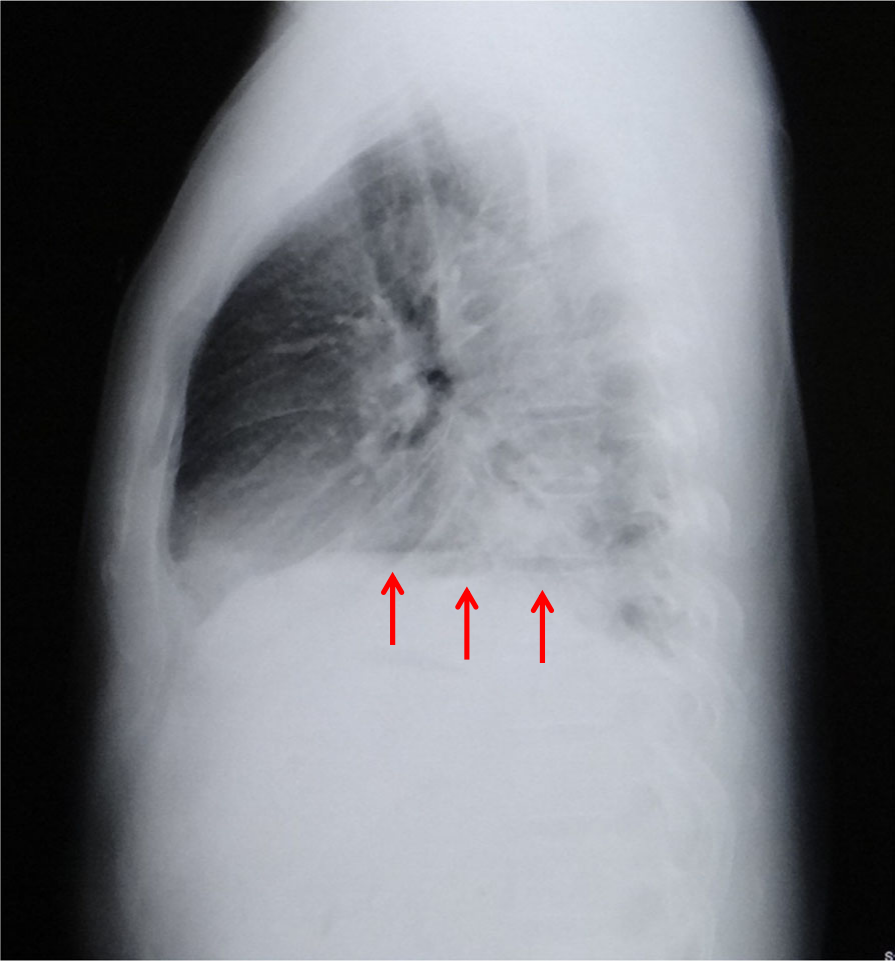
Chest X-ray of the lateral view (seated position) on arrival in case 1. Several areas of free-air (arrows) just below the diaphragm are evident

**Fig. 2:**
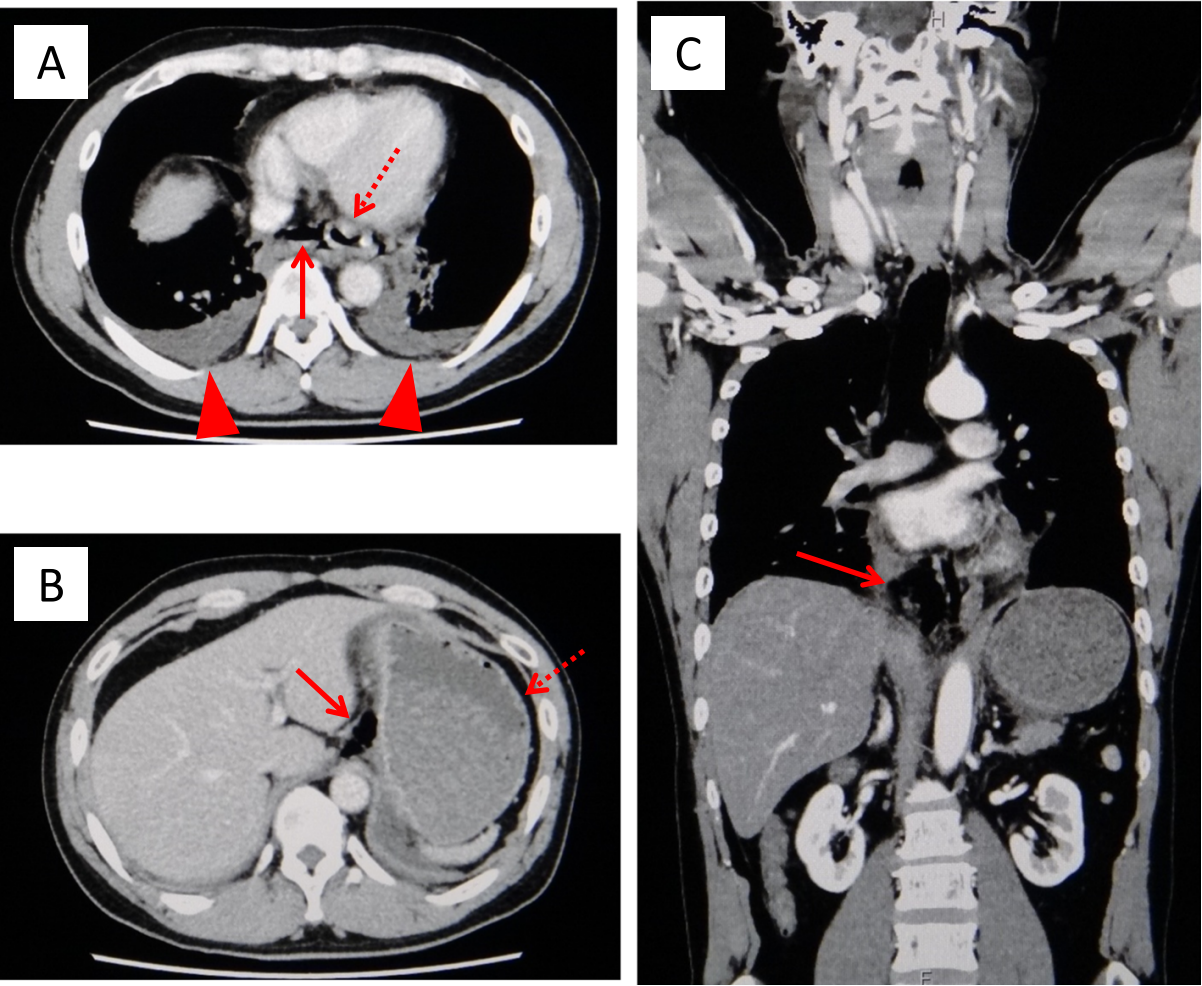
Contrast-enhanced CT on arrival in case 1 (**a**, **b** transverse section; **c** sagittal section). Free-air (arrows) around the distal esophagus, the lesser curvature of the stomach, and the para-aortic area of the abdomen is observed. Bilateral pleural effusion (arrowheads), and the dilated esophagus and stomach (dotted-line arrows) are also observed

**Fig. 3:**
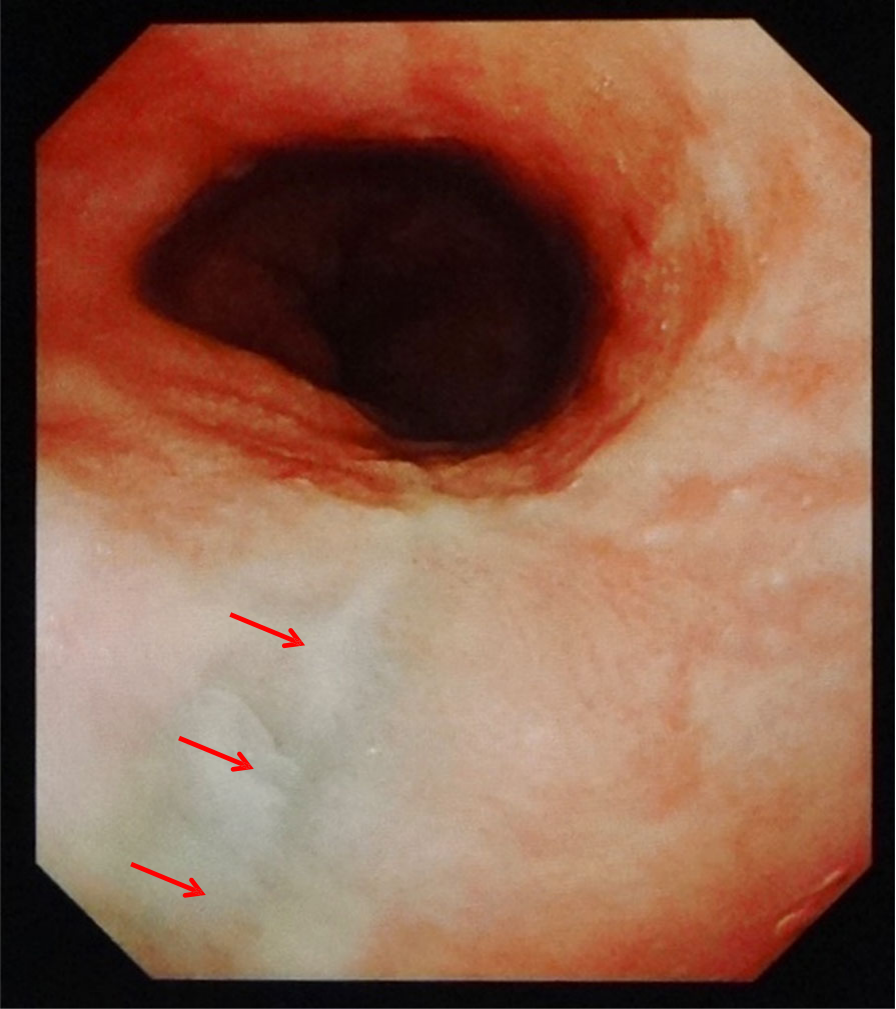
Upper endoscopy performed 98 days after the first visit in case 1. A scar (arrows) after the suture is observed on the left posterior wall of the distal esophagus 38–41 cm from the incisors

A 45-year-old man with a history of hypertension, dyslipidemia, and habitual drinking visited our hospital with complaints of severe chest pain, back pain, bilateral shoulder pain, and vomiting. He fell ill after heavily consuming alcohol for a day. Three and a half hours prior to his arrival, he experienced discomfort in his chest followed by vomiting. Two hours and 20 min prior to his arrival, he felt nauseated, describing a feeling of sudden stiffness throughout his entire upper body. On arrival, he was alert, his blood pressure was 139/98 mmHg, pulse rate was 64/min and regular, body temperature was 36.5 °C, respiratory rate was 20/min, and oxygen saturation was 95%. He was 173 cm tall and weighed 73.1 kg. No abnormalities other than cold sweat and epigastric tenderness were noted on physical examination. Chest radiography of the lateral view (seated position) showed several areas of free-air just below the diaphragm (Fig. 1). Contrast-enhanced computed tomography (CT) showed free-air, bilateral pleural effusion, and a dilated esophagus and stomach (Fig. 2). Based on these findings, an esophageal rupture with mediastinitis was the definitive conclusion and he was thus diagnosed with Boerhaave syndrome. A subsequent esophagogram further confirmed the diagnosis. Nine hours after the onset of the rupture, an emergency operation was performed as follows. Through a left thoracoabdominal incision, closure of the perforation and drainage of both the mediastinum and thoracic/abdominal cavities were performed in succession. The penetrating laceration was approximately 3 cm in length vertically on the left posterior wall of the distal esophagus and was patched with the patient’s omentum. Although the patient had a complication of anastomotic insufficiency on the 16th day, it was ultimately resolved. The patient was then administered a liquid diet on the 35th day, and he was discharged on the 41st day. Upper gastrointestinal endoscopy was performed on the 98th day, and a scar due to the suture was observed in the same location as the previous perforation (Fig. 3).

### Case 2

**Fig. 4:**
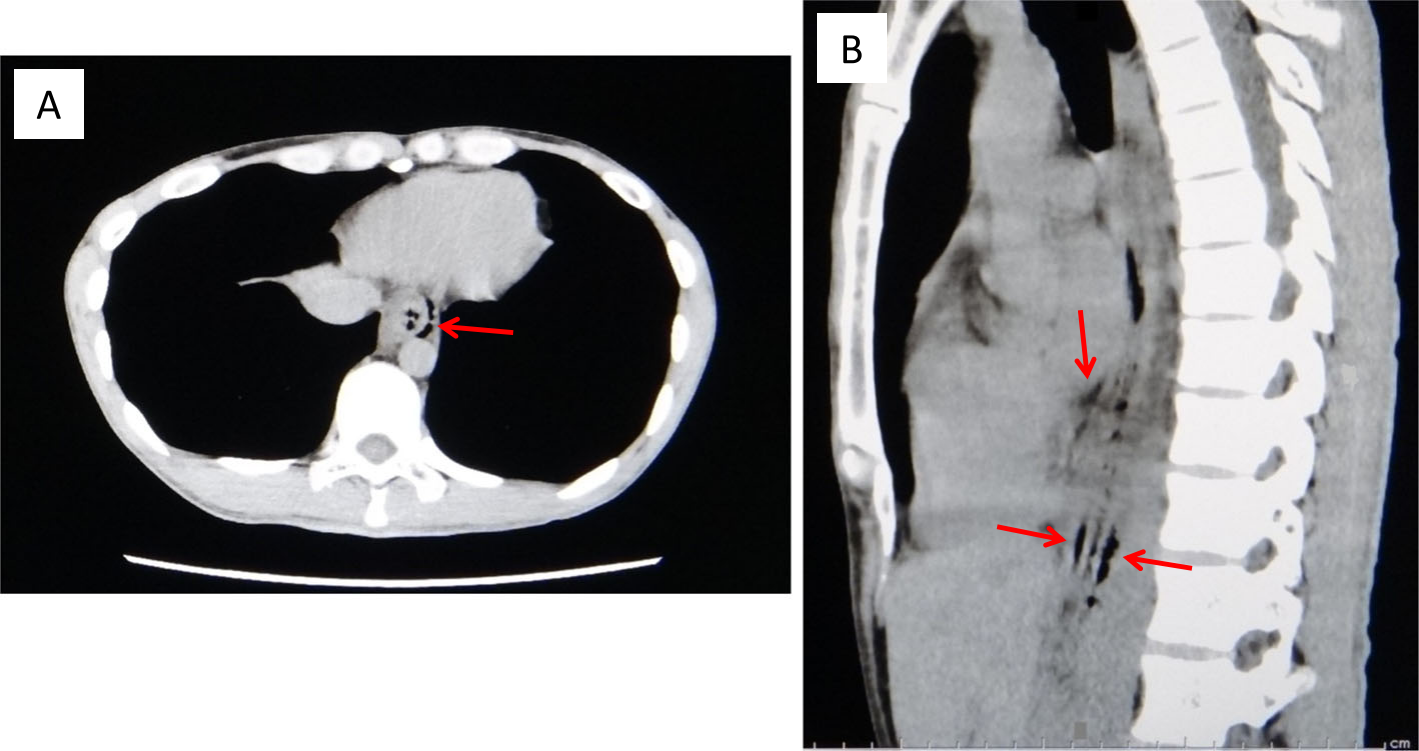
Plain CT on arrival in case 2 (**a** transverse section; **b** sagittal section). Small free-air (arrows) is observed in the mediastinum around both the EGJ and the boundary between the middle and lower thoracic esophagus

**Fig. 5:**
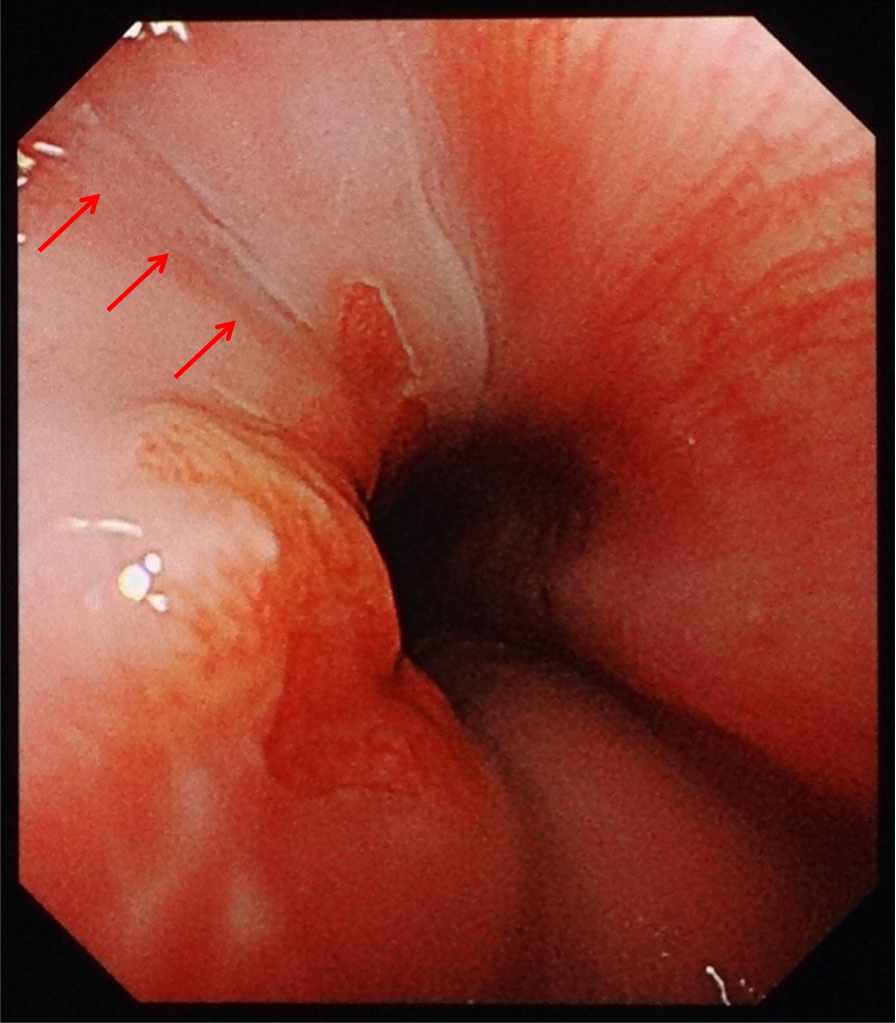
Upper endoscopy performed 9 days after the first visit in case 2. A linear scar (arrows) is observed on the left wall of the distal esophagus, including the EGJ

A 27-year-old previously healthy man visited our hospital with complaints of vomiting, hematemesis, pyrosis, severe chest pain, and epigastralgia. He had been drinking alcohol heavily until 14 h prior to his arrival. Six hours prior to his arrival, he experienced epigastralgia and nausea, followed by retching and diarrhea. Three and a half hours prior to his arrival, he started experiencing pyrosis and intermittent chest pain and had an episode of hematemesis. On arrival, he was alert, his blood pressure was 110/62 mmHg, pulse rate was 96/min and regular, body temperature was 37.4 °C, respiratory rate was 18/min, and oxygen saturation was 97%. He was 172.3 cm tall and weighed 53.5 kg. Epigastric tenderness and retrosternal pain with forced respiration were noted on physical examination. Although chest radiography showed no findings, plain CT showed small areas of free-air in the mediastinum (Fig. 4). At first, Mallory–Weiss syndrome was suggested because of hematemesis, but CT suggested Boerhaave syndrome. No hematemesis recurred during his hospitalization, and his condition was followed up on via esophagogram and CT. Upper endoscopy was performed on the ninth day, and a linear scar was observed on the left wall of the distal esophagus, including at the esophagogastric junction (EGJ) (Fig. 5). The injury healed after conservative treatment that included rest, no oral consumption, and preventive antibiotics. He was discharged on the 11th day.

## Discussion and conclusions

A comparison of the two cases is shown in Table 1. Both patients were equally distressed on arrival, but the clinical course was more complicated in case 1 than in case 2. The diagnoses of both cases were conventional and symptomatic, involving vomiting, pain in the lower thorax, and mediastinal emphysema [4].

**Table 1:** Comparison of the reported cases

	Case 1	Case 2
Age (years)	45	27
Physique (body mass index)	24.4	18.0
Symptoms	Vomiting, chest pain, back pain, bilateral shoulder pain	Vomiting, chest pain, epigastralgia, hematemesis, pyrosis
Severity	Distressed	Distressed
Trigger	Excessive drinking from the previous day	Excessive drinking from the previous day
Hematemesis	–	+
Operation	+	–
Location	On the left posterior wall of the distal esophagus	On the left wall of the distal esophagus
First endoscopy	Day 68	Day 9
Period of hospitalization	41 days	11 days
Estimated time from onset to rupture	1.2 h	2.5 h
Estimated time from rupture to operation room	9 h	(No operation)

Esophagography, upright chest radiography, and chest CT are useful for accurately diagnosing Boerhaave syndrome [5, 6]. In cases similar to case 1, in which patients present with major hydropneumothorax or pneumomediastinum, chest radiography is more appropriate. If such presentations are minor, as in case 2, CT is preferred, owing to recent improvements in imaging quality. In case 2, conservative treatment revealed that if inflammation due to the rupture is localized in the mediastinum, non-operative choices might be successful [3]. Radiological modalities, however, are not useful for the diagnosis of Mallory–Weiss syndrome. In cases where Boerhaave syndrome is unlikely, endoscopy is recommended for the diagnosis and concurrent treatment, as the source of bleeding in Mallory–Weiss syndrome is difficult to detect even with contrast-enhanced imaging studies.

Boerhaave syndrome occurs owing to the lack of coordination between the upper and lower esophageal sphincters, resulting in a transmural tear of the distal esophageal wall due to an increased intragastric pressure transmitted to the esophagus during vomiting [7]. In both presented cases, the left wall of the distal third of the esophagus was torn. This is a distinctly common localization due to the thin muscular layer, nerve and vascular entry points, and lack of support for the surrounding connective tissue [7].

Several reports have described diagnostic errors in which esophageal rupture was diagnosed as Mallory–Weiss syndrome [8–10]. Commonly, massive hematemesis with less pain indicates Mallory–Weiss syndrome, while minimal hematemesis with more pain indicates Boerhaave syndrome, despite both disorders presenting with an onset of vomiting after heavy drinking. Hematemesis was observed in case 2, but the endoscopic findings showed a scar only on the esophagus, not on the stomach; moreover, Mallory–Weiss lesions were not observed at all (Fig. 5).

The etiologies of both Boerhaave and Mallory–Weiss syndromes are similar, both result from abnormally elevated intraluminal cardio-esophageal pressure during vomiting. Prior studies have mentioned the relationship between the two syndromes [11–17], with some even identifying Boerhaave syndrome as an extension of Mallory–Weiss syndrome [12–17]. However, this may be disputed for two reasons: the differences in the common sites (left distal esophagus vs. gastric cardia) and the scarcity of reported transitional cases. Our review of accessible transitional cases [14–17]—except for one case [17] that might have a true transitional lesion—showed that those cases [14–16] not only had several lacerations that extended into the submucosa of the gastric cardia but also a separate single laceration through all layers of the distal esophagus. Thus, the two syndromes are distinct, and it is worth considering that many transitional cases are situations in which both syndromes occurred independently in the same individual. Regarding pathophysiology, Mallory–Weiss lacerations are caused by repeated retching and primarily involve the part of the gastric cardia that includes the EGJ. Boerhaave’s lacerations, on the other hand, may occur all at once in the weakest part of the esophageal wall, as the muscular layer initially tears before the mucosal layer when an explosive force is applied [18].

In summary, we report two cases of Boerhaave syndrome with different severities. Severity may vary greatly among patients with this disorder. Plain CT can quickly and easily detect any severity of the syndrome. Further, it can differentiate Boerhaave from Mallory–Weiss syndrome; thus, it is recommended as a preliminary test, if feasible. Although the initial cause of Boerhaave syndrome is the same as that of Mallory–Weiss syndrome, the nature of the two syndromes is distinctive and correct diagnosis is essential for positive patient outcomes.

## Data Availability

Not applicable

## Abbreviations

CT: Computed tomography
EGJ: Esophagogastric junction
